# A randomised clinical trial of a digital self-management package for people with interstitial lung disease: the REBUILD-SM protocol

**DOI:** 10.1186/s12931-025-03442-z

**Published:** 2025-12-05

**Authors:** Carly Barton, Mariana Hoffman, Narelle S. Cox, Laura M. Glenn, Joanna Y. T. Lee, Ingrid Cox, Nicole S. L. Goh, Lauren K. Troy, John Mackintosh, Daniel C. Chambers, Ian N. Glaspole, Val Gebski, Anthony Keech, Andrew Palmer, Liliana Laranjo, Luke D. Knibbs, Yuben Moodley, Mark Brooke, Anne E. Holland, Tamera J. Corte

**Affiliations:** 1 Department of Respiratory and Sleep Medicine, Royal Prince Alfred Hospital, Sydney, NSW Australia; 2 The University of Sydney School of Medicine (Central Clinical School), Sydney, NSW Australia; 3 NHMRC Centre of Research Excellence in Pulmonary Fibrosis, Sydney, Australia; 4 School of Translational Medicine, Monash University, Melbourne, VIC Australia; 5 Institute for Breathing and Sleep, Melbourne, VIC Australia; 6 Menzies Institute for Medical Research, The University of Tasmania, Hobart, Australia; 7 Faculty of Medicine, University of Melbourne, Melbourne, VIC Australia; 8 Department of Respiratory and Sleep Medicine, Austin Health, Melbourne , VIC Australia; 9 Department of Thoracic Medicine, The Prince Charles Hospital, Brisbane, QLD Australia; 10 Faculty of Medicine, University of Queensland, Brisbane, QLD Australia; 11 Department of Respiratory and Sleep Medicine, Alfred Health, Melbourne, VIC Australia; 12 NHMRC Clinical Trials Centre, The University of Sydney, Sydney, NSW Australia; 13 Westmead Applied Research Centre, University of Sydney, Sydney, NSW Australia; 14 School of Public Health, The University of Sydney, Sydney, NSW Australia; 15 Public Health Research Analytics and Methods for Evidence, Public Health Unit, Sydney Local Health District, Sydney, NSW Australia; 16 Centre for Respiratory Health, University of Western Australia, Perth, WA Australia; 17 Stem Cell Unit, Institute for Respiratory Health, Perth, WA Australia; 18 School of Medicine, University of Western Australia, Perth, WA Australia; 19 Dept of Respiratory Medicine, Fiona Stanley Hospital, Perth, WA Australia; 20 Lung Foundation Australia, Brisbane, Australia QLD; 21 Department of Physiotherapy, Alfred Health, Melbourne, VIC Australia

**Keywords:** Interstitial lung disease, Self-management, MHealth, Mobile health, Applications, Self-monitoring

## Abstract

**Background:**

Interstitial lung disease (ILD) has a profound impact upon health-related quality of life (HRQoL) and mortality. Patients with ILD have identified their desire for tools to improve self-management. This study compares the clinical efficacy and cost-effectiveness of delivering an ILD-specific self-management package through a mobile health smartphone application (the REBUILD app), with standard care. It also evaluates the barriers and facilitators to adoption of the intervention in clinical practice.

**Methods:**

This is a prospective, multicentre, randomised control trial (RCT) with embedded economic and implementation evaluations, with recruitment via ILD clinics at four Australian tertiary hospitals. Participants are allocated 1:1 to intervention and control groups, with randomisation stratified by disease severity, diagnosis, and site. The intervention group receives the self-management package and four phone health coaching sessions over 12 weeks. The control group receives standard care and four phone calls to control for attention. Outcomes will be measured at 12, 26 and 52 weeks. The primary outcome is change in HRQoL at 12 weeks, measured by the King’s Brief Interstitial Lung Disease questionnaire.

**Discussion:**

Enhanced self-management has been associated with better HRQoL in several chronic respiratory conditions, however its effect in people with ILD is not known. This is the first multicentre RCT to evaluate the effect of a digital self-management intervention delivered through a smartphone app in people with ILD. If successful, this intervention has the potential to enhance access to self-management support for people with ILD.

**Trial registration:**

This project was prospectively registered with the Clinical Trial Registry (NCT06122233||https://www.clinicaltrials.gov//{08/11/2023}}.

## Background

The interstitial lung diseases (ILDs) are a group of chronic inflammatory and fibrotic lung disorders that share the symptomatology of worsening breathlessness, cough, impaired exercise tolerance, anxiety, and often extremely poor health-related quality of life (HRQoL) [1]. A global ILD prevalence of 6.3 to 71 per 100,000 and incidence of 1–31.5 per 100,000 person years have been reported, with estimates varying regionally due to heterogeneity of ILD categorisation methods [2]. The most prevalent form of fibrotic ILD is idiopathic pulmonary fibrosis (IPF), a severe and progressive lung disorder with median survival of only 2–5 years from diagnosis if left untreated [3]. Crude and age-standardised estimates of IPF incidence in Australia were recently reported as 10.4 and 11.2 per 100,000 population, respectively, while estimated age-adjusted mortality was 6.3 per 100,000 population – worse than for many cancers [4]. In Australia, an estimated $299 million is spent on IPF healthcare annually [5]. 

Idiopathic pulmonary fibrosis comprises approximately 30% of ILD in local and international cohorts [6, 7]. Increasing evidence shows other forms of fibrotic ILD share overlapping pathogenic and prognostic features, with common therapeutic targets. The antifibrotic drugs, nintedanib and pirfenidone, slow the deterioration of progressive fibrotic ILD [8–11], but do not improve HRQoL and in some cases, may contribute to worsening HRQoL through adverse effects [8, 9]. Holistic management strategies for people with ILD are desperately needed.

The 2019 Australian *National Strategic Action Plan for Lung Conditions* prioritised measures to support people with ILD, including improving self-management capabilities [12]. Self-management is an individual’s ability to manage the physical and psychosocial sequelae of living with a chronic condition, including symptoms, treatment, and lifestyle modifications, and implies a proactive involvement in one’s health [13]. Self-management has an established role in chronic obstructive pulmonary disease (COPD), asthma and cystic fibrosis [14, 15] and has been associated with improved HRQoL in these conditions. However, it is a relatively new concept in ILD with limited published data, although recognition of its importance is growing [16–18]. People with ILD report feeling a high level of responsibility for maintaining their health, and a strong desire for self-management [18–20]. An ILD self-management package was developed in partnership with ILD healthcare professionals and consumers, [17] with 12 components of a self-management program identified and confirmed by an independent Delphi consensus [21]. A recent randomised control trial (RCT) evaluated the feasibility of this package with positive results, [22] and an additional module, ‘Managing Weight and Nutrition,’ was incorporated based on participant feedback.

Mobile health or mHealth apps can facilitate self-management, leveraging widely available technology for broad reach. To date, mHealth apps used to support self-management have demonstrated positive effects on HRQoL in asthma, COPD, and cystic fibrosis, [23–25] however their impact in patients with ILD is unknown. Together with consumers engaged through the Centre of Research Excellence for Pulmonary Fibrosis (cre-pf.org.au) Consumer Advisory Group, our team designed and developed the REBUILD app, with a recent pilot study showing high user engagement and satisfaction with the intervention [26]. 

## Methods

### Study design and setting

The REBUILD-SM trial is a multicentre parallel-group RCT with embedded implementation and economic evaluations recruiting 400 people with ILD. It is expected to run from 2024 to 2027 and be conducted at four Australian tertiary hospitals currently participating in the Australasian ILD Registry (AILDR). Participant enrolment commenced in June 2024 with anticipated completion by December 2026.

### Participants

People with fibrotic ILD are eligible if they (i) are ≥ 18 years of age; (ii) can provide informed consent; (iii) have been on stable ILD treatment for the month preceding enrolment; (iv) own or have access to a smartphone or tablet to download the REBUILD app to; (v) have access to email; (vi) are able to understand written and spoken English; and (vii) have sufficient digital literacy as quantified by the ability to use a mobile phone, download new apps, and enter keywords into a search box [27]. Exclusion criteria includes (i) acute ILD exacerbation in the month preceding enrolment; (ii) participation in pulmonary rehabilitation at enrolment or; (iii) death or transplant expected within the 12 week intervention period.

### Recruitment and consent

Participants are recruited from ILD clinics at four sites and through the AILDR. Potential participants are approached by a healthcare professional in person or via phone and given information about the study. AILDR participants are sent a recruitment letter via post or email. A member of the research team will contact interested potential participants and those wishing to enrol will download the REBUILD app and complete the consent electronically.

### Randomisation, concealment of allocation and blinding

Participants are randomised via the app 1:1 to intervention or control group for the 12-week intervention period. Permuted block randomisation, stratified by (i) diagnosis of IPF or not; (ii) FVC of ≤ 50% or ≥ 51%; and (iii) study centre will occur upon entry of these factors. Participants failing to enter this data will not be randomised. The nature of the intervention makes blinding of participants and healthcare professionals delivering the intervention impractical; however, endpoint assessment will be blinded for outcome assessors and data analysts using a Prospective Randomised Open Blinded Endpoint (PROBE) [28] approach. This study is designed according to SPIRIT guidelines [29] and will be reported according to CONSORT standards [30]. 

### Intervention – self-management package

In addition to standard care, the intervention group will receive access to the ILD self-management content, four health coaching support phone calls, and the full version of the REBUILD app including educational resources. While the intervention is designed to improve the ability of participants to self-manage their disease, it is not intended to replace clinical care for either group. Both groups still attend their regular ILD clinic reviews and are managed according to usual care pathways by their treating team.

**Fig. 1:**
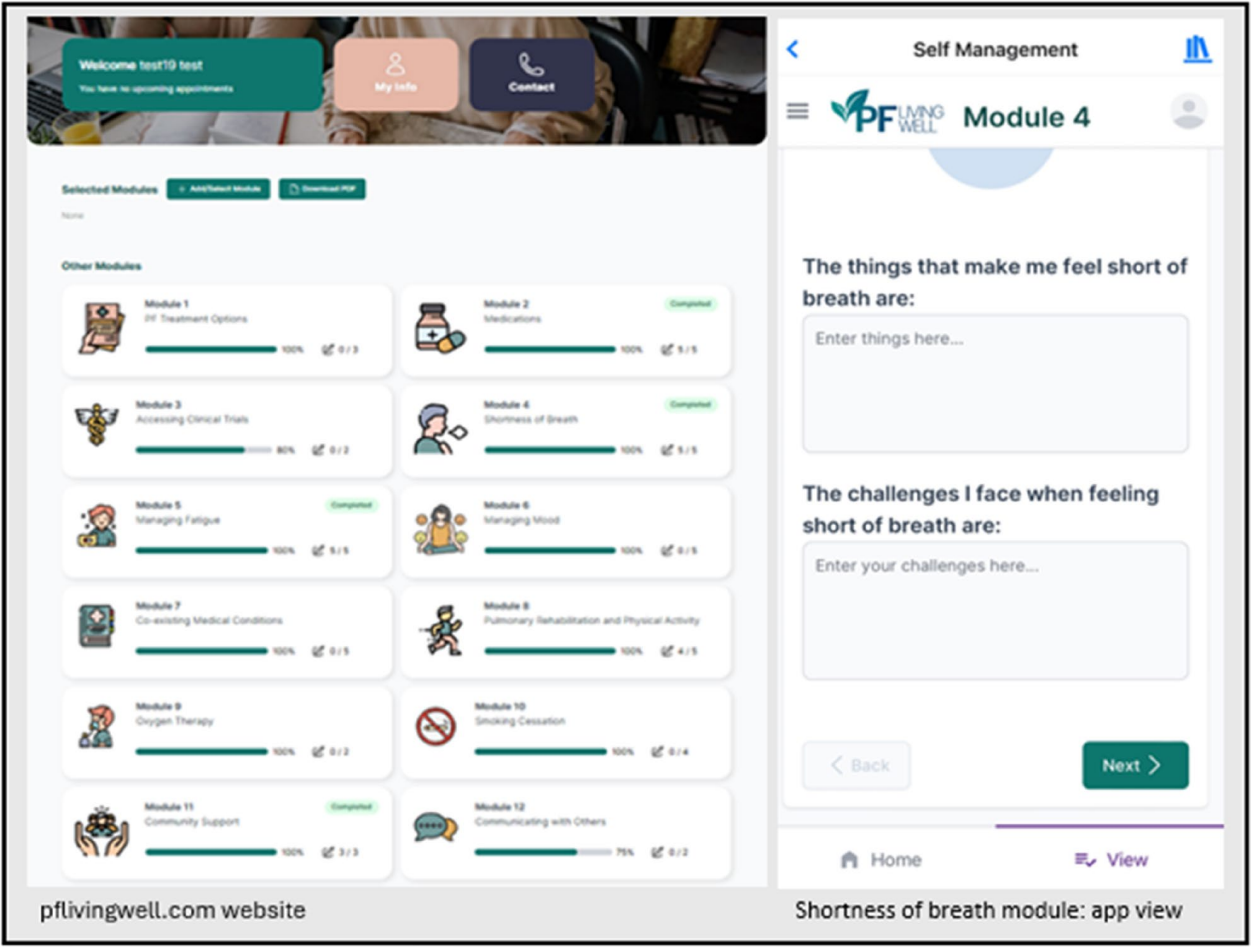
REBUILD-SM self-management modules

The intervention group can access the self-management package via *pflivingwell.com* and the REBUILD app (Fig. 1). The content includes 13 modules, organised by topic, each providing key information and embedded hyperlinks to relevant website, factsheet, booklet, video, and webinar resources vetted by our research team. The intervention is designed to improve self-management through information and activities related to goal setting, symptom tracking, self-reflection and problem-solving, compared with self-monitoring through the app alone [22]. Prior to accessing the self-management content, the intervention group complete an online introduction to identify which modules best align with their personal concerns and goals. Participants are then encouraged to review these modules throughout the intervention period.

### Health coaching support calls

The intervention group will receive health coaching via phone or video call at 4 time points (week 1, 2, 3 and once between week 6 and 12) during the study period, with the aim of promoting engagement with the self-management modules. Calls will be made by an ILD healthcare professional who, throughout the intervention period, may provide support such as (i) guidance in identifying appropriate modules; (ii) assisting the participant to set health-related goals and identify strategies to achieve these; (iii) monitoring progress; (iv) addressing challenges; and (v) providing feedback or clinical recommendations. These calls also provide an opportunity for the healthcare professional to assess the participants’ understanding and tailor support accordingly. The outcomes of each session will be documented.

### The REBUILD app

**Fig. 2:**
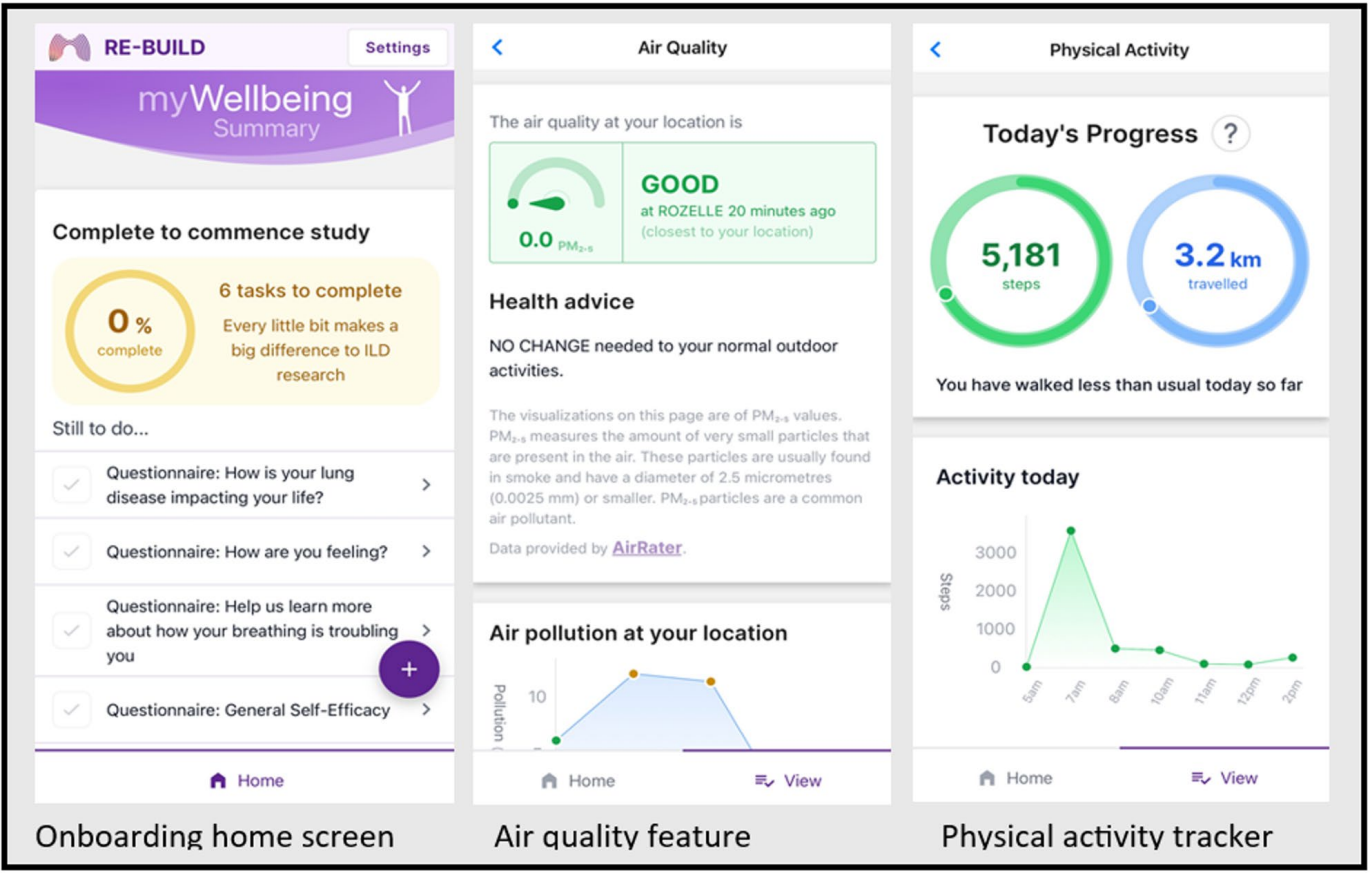
REBUILD app screens

The REBUILD app is designed to be a self-monitoring tool for both intervention and control group participants and is available for iOS and Android devices. It includes four sections: ‘Measurements’; ‘Your Tracking’; ‘Your Diaries’; and ‘Other Information.’ Various app screens are shown in Fig. 2. Participants in both groups will receive all sections, however only the intervention group will be able to view all components of ‘Other Information.’

#### Measurements

In this section, participants can enter their lung function test values (FVC and DLCO) and resting blood oxygen saturation (Sp0_2_) levels after obtaining this information from their ILD clinic assessment. Weight can also be recorded here, and participants are instructed to update this section when new results become available. Values are displayed graphically in the app to show trends over time.

#### Your tracking

Participants can opt to receive local air quality and step count information in real-time. The air quality feature displays location-specific air quality data sourced from www.airrater.org. This data includes PM_2.5_ (particulate matter with a diameter ≤ 2.5 micrometres) readings and health advice from the relevant state or territory health department, e.g., reduce outdoor physical activity if symptoms such as cough or shortness of breath arise when air quality is ‘fairly good’; and to stay inside with windows and doors closed if it is ‘poor.’ PM_2.5_ readings at the participant’s location are recorded throughout the trial. To track physical activity, the REBUILD app syncs with the Apple Health or Google Fit apps on the participants device, enabling step count and distance travelled to be displayed in-app for motivation.

#### Your diaries

Participants can record their medications, side effects, and supplemental oxygen use here and are encouraged to keep this information updated. The intervention group can also access the self-management content here.

#### Other information

Participants of both groups can record data such as ILD diagnosis, comorbidities and smoking history here. Phone and email technical support contact details are in this section and visible to all participants. In addition, the intervention group can access links to ILD-related educational resources, including videos, PDFs, and websites, as well as a one-way messaging inbox that can be used by the study team to alert participants to clinical trials of potential relevance to them. These components are not visible to the control group.

### Control group

The control group receive the same standard of clinical care as the intervention group, including regular ILD clinic reviews, meaning the medical management of both groups is the same. The control group also receive access to a reduced capability or ‘lite’ version of the REBUILD app for remote data collection and self-monitoring only. The lite version of the app does not include access to the educational materials or the self-management content but does include the physical activity and air quality features described above. To control for the effects of attention, participants in the control group will receive calls at the same frequency as the intervention group, however these calls will be general in nature with no health advice provided. Calls will be completed by site study coordinators, who will offer technical support only. Participants seeking health advice will be directed to their usual care providers.

**Fig. 3:**
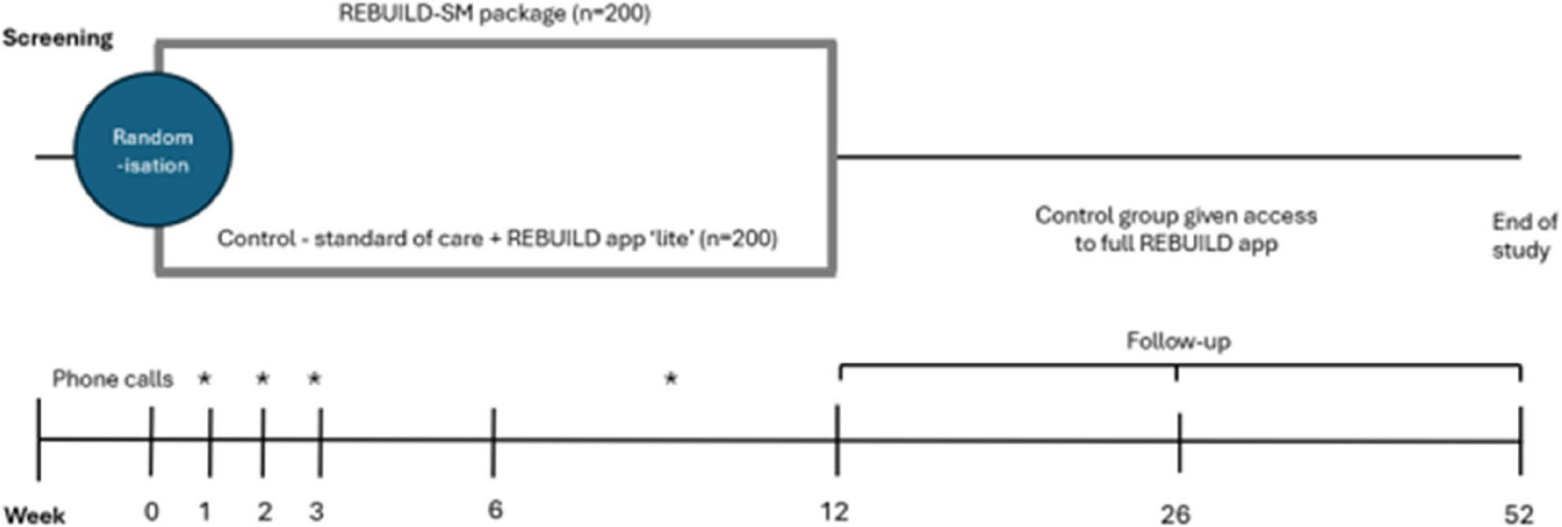
Study timeline

At week 26, participants in the control group will receive access to the full version of the REBUILD app (Fig. 3) on ethical grounds, however this will not include access to the self-management package or the phone support provided in the intervention. This difference will be accounted for during the final data analysis.

### Aims and outcomes

This study aims to compare the clinical efficacy and cost-effectiveness of the self-management package delivered through the REBUILD app to standard clinical care; and to understand the barriers and facilitators to adoption of the intervention. The study team hypothesise that REBUILD-SM will (1) improve HRQoL, symptoms, anxiety, self-efficacy, and physical activity in people with ILD; (2) be a cost-effective intervention compared to standard care; and (3) be implementable in clinical practice.

The primary outcome is change in HRQoL measured by change in King’s Brief Interstitial Lung Disease (K-BILD) questionnaire score at 12 weeks. The K-BILD has been chosen as a valid, ILD-specific measure of HRQoL with demonstrated responsiveness to interventions; [31] and this timepoint was selected based on evidence showing change in physiological variables at 12 weeks to be predictive of mortality and disease progression in IPF patients [32]. Pulmonary rehabilitation (commonly lasting between 8 and 12 weeks) has also demonstrated efficacy in improving short-term HRQoL in people with IPF, [33] suggesting that 12 weeks will be sufficient to observe a treatment effect.

The secondary outcomes are as follows (see also Table 1):

- Change in K-BILD score from baseline at week 26 and 52; and change in EQ-5D-5L score from baseline at week 12, 26 and 52
- Change in General Self-Efficacy Scale (GSE) score from baseline at week 12, 26 and 52
- Change in Dyspnoea-12 score from baseline at week 12, 26 and 52
- Change in Hospital Anxiety and Depression Scale (HADS) score from baseline at week 12, 26 and 52
- Change in physical activity level from baseline at week 12 and 52
- Uptake or changes to ILD treatments, including clinical trial enrolment at week 12, 26 and 52
- Acceptability of the REBUILD app, evaluated utilising the mHealth App Usability Questionnaire (MAUQ) score at week 12, 26, and 52; and app analytics, including frequency the app and its individual features are accessed and used, total time of use and percentage of complete data entered
- Change in eHealth and Literacy Scale (eHEALS) score from baseline at week 12 and 52
- Autonomy, which will be evaluated using the Healthcare Climate Questionnaire (HCCQ) at week 12

**Table 1:** Study endpoints

**Data collection**	**Baseline**	**Week**			
		**12**	**26**	**38**	**52**
Demographic and clinical information	**X**		**X**		**X**
HRQoL (K-BILD & EQ-5D-5L questionnaire)	**X**	**X**	**X**		**X**
GSE	**X**	**X**	**X**		**X**
Dyspnoea-12	**X**	**X**	**X**		**X**
HADS	**X**	**X**	**X**		**X**
7 days physical activity monitoring (StepWatch)	**X**	**X**			**X**
Uptake or changes to treatments for ILD	**X**	**X**	**X**		**X**
All-cause hospitalisation / ED visits in last 12 months	**X**		**X**		**X**
Cost Diary	**X**	**X**	**X**	**X**	**X**
MAUQ		**X**	**X**		**X**
eHEALS	**X**	**X**			**X**
HCCQ		**X**			

### Data collection

Study endpoints are completed using the REBUILD app, with push notifications informing participants when they have tasks to complete. Reminders will be sent at 24 and 48 h to encourage data completion, followed by a reminder phone call from sites if data is still pending at 72 h. An additional phone call will be completed if the endpoints remain outstanding after 5–7 days. Step count over a 7-day period will be measured at baseline, week 12 and 52 using the StepWatch activity monitor (Modus Health, Washington DC, USA), a reliable and validated tool used in patients with chronic lung disease [34]. Information on Emergency Department (ED) presentations and hospitalisations as well as uptake and changes to ILD treatment and clinical trial enrolment will be gathered from the participants’ electronic healthcare record.

### Economic evaluation

Assessed using a cost diary administered via Research Electronic Data Capture (REDCap) link at baseline and week 12, 26, 38 and 52, as the complexity and length of the cost diary makes it unsuitable for in-app completion. Additional information will be drawn from state-base hospital records, Pharmaceutical Benefits Scheme (PBS) and Medicare Benefits Schedule (MBS) data, with existing approved linkage between these and the AILDR.

### Implementation evaluation

To underpin any future implementation, we aim to understand the barriers and facilitators to adoption of REBUILD-SM by people with ILD as well as healthcare professionals using the following methods.

#### Participant experience

Following completion of the 12-week intervention, semi-structured telephone interviews will be undertaken with a purposive sample of intervention group participants. These interviews aim to facilitate understanding of the patient experience and will be conducted by a researcher not involved in delivering the intervention. A sampling framework will be used to select participants and interviews will be recorded and transcribed verbatim. An interview guide will be used, and qualitative data obtained from these interviews will be analysed using the principles of grounded theory [35]. Recruitment will continue until data saturation is achieved.

#### Healthcare professional experience

Qualitative interviews will be undertaken with healthcare professionals involved in the trial during the last 26 weeks of recruitment at their site. An interview guide will be used, and interviews will be completed via phone, recorded and transcribed verbatim. The aim of these interviews is to understand the healthcare professionals’ experience of REBUILD-SM and the use of a technology-based intervention to deliver the intervention. The Theoretical Domains Framework [36] will be used to understand behaviours that function as barriers or facilitators to the implementation of the intervention into clinical practice.

#### Translational and behaviour change success

This will be assessed using the RE-AIM framework (Table 2) which aims to maximise implementation and integration of evidence-based health care interventions. This data will inform development of a Self-Management Toolkit, in partnership with key stakeholders, to accompany the digital package and facilitate adoption into local ILD services.

**Table 2:** Implementation evaluation outcomes according to RE-AIM framework

RE-AIM element	Outcomes Assessed
Reach	Percent of eligible individuals who participate Characteristics of participants (age, sex, postcode, disease severity, HRQoL, eHEALS)
Effectiveness	Intervention completion (> 75% planned telephone sessions delivered) Number of self-management goals set (intervention group) Clinical outcomes, number of people who take up pulmonary rehabilitation, healthcare utilisation, adverse events Patient experience (semi-structured interviews)
Adoption	Intervention uptake (number who accessed the app on at least one occasion; number who attended start-up phone consultation and set a goal – intervention group) Frequency of app access (both groups) Characteristics of staff delivering intervention; staff time to get participants onto the app; number of occasions staff need to assist participant to get onto the app Health care professional experience (semi-structured interviews), clinical partner involvement
Implementation	Number of self-management modules accessed Modules delivered Adaptations made to intervention Staff time (from phone call record, both groups)
Maintenance	Individual: Use of REBUILD-SM, clinical outcomes at 6 months. Self-efficacy score as a marker for perceived self-management skills Organisation: Intent to continue using REBUILD-SM at 6 months post-trial completion, local funding models, modifications made

## Statistical considerations

### Sample size justification

A sample size of 400 subjects randomised equally between groups will provide at least 82% power with 95% two-sided confidence to identify a five K-BILD unit improvement from the usual care mean of 55.3 (SD 15.6), with a 5-unit increase considered the minimum clinically important difference [31]. Assuming a pooled SD of 6.5% this offers >95% power to identify a statistically significant difference between groups of 5 points in K-BILD score (change of + 2 in control and + 7 in active arm), allowing for up to 15% of randomly missing endpoint data. Even for a difference of 3 points in Z-score, the study will similarly offer 95% power. This sample size allows for a modest dropout rate of 10%.

Participant mortality should not negatively impact the primary endpoint data as participants expected to die during the 12 week trial period are excluded. Randomisation will be performed using the method of minimisation stratified by (i) FVC: ≤50% or ≥ 51%; (ii) IPF: yes/no; and (iii) treating site.

### Data analysis

#### Quantitative analysis

The primary endpoint is the difference from baseline in K-BILD scores at 12 weeks according to the intention-to-treat principle. Primary analysis will evaluate whether these differences are larger in the intervention group compared to the control group using 2-sided tests at a 5% level of significance. Key secondary analyses will evaluate the proportion of those with 12-week K-BILD scores above and below 50 and whose K-BILD has increased from baseline by at least 5 units. Additional analyses of secondary endpoints will be performed using standard statistical tests as appropriate.

Associations between 12-week K-BILD score and key baseline variables will be evaluated. Acceptability outcome measures will be reported using the MAUQ. Feasibility will be measured as adherence to the clinical trial (defined as at least 80% completion of data questionnaires). Participants will be considered adherent to the intervention if they receive at least 75% of planned self-management phone calls and access the self-management website on at least one occasion. Percentage of completed modules will also be reviewed, as well as weekly time spent using the app during the study period. The primary and key secondary outcomes will be evaluated in subjects who remain adherent to the intervention in a per protocol analysis.

Analysis of the effect of the REBUILD-SM intervention in key subgroups will also be performed. These subgroups include FVC ≤ 50% or FVC ≥ 51%; IPF yes/no; time since ILD diagnosis < 12 months or ≥ 12 months; and age < 65 or ≥ 65 years.

Modus Research software will be used to retrieve StepWatch data. Participants will be asked to record their activity for 7 consecutive days, and up to 5 complete days of data will be used, with the first and last days of the activity period discarded due to incompleteness. Days with less than 200 steps or less than 24 h of recording time will also be discarded. Average steps per day over all included days will be calculated and difference in measures of physical activity between baseline and week 12 will be compared between groups.

#### Economic evaluation

Within trial cost effectiveness and cost-utility analyses will be conducted, and trial outcomes will be extrapolated to a 1, 2 and 5-year time horizons using a disease simulation model. Mean total costs (direct and indirect) will be assessed for both control and intervention groups. For modelling purposes beyond the trial period, mean total annual costs for ILD-related and all healthcare utilisations will be estimated, and appropriate discount rates applied. The mean effect between baseline and 52 weeks of both intervention and control groups will be assessed by measuring the change in primary outcome measure, the K-BILD (for cost-effectiveness analysis) and change in health state utility values and subsequently quality-adjusted life-years (QALYs) derived from the EQ-5D-5L questionnaire (for the cost utility analysis). Appropriate discount rates will be applied for modelling purposes beyond the trial period. The incremental cost effectiveness (ICER)/incremental cost utility (ICUR) ratio will be assessed and compared between each group. To address any uncertainty around estimates, uncertainty, sensitivity, and subgroup analyses will be conducted. Disease simulation modelling will then be conducted to project within-trial outcomes beyond the trial period at 1, 2 and 5-year time horizons. Using Markov simulation modelling, we will determine and compare total time horizon costs, QALYs, and future ICER/ICUR between intervention and control groups.

### Data management

Only deidentified information is collected and all data is encrypted prior to transmission. Data collected by the app is stored in the cloud using Amazon Web Services (AWS, Sydney NSW). Completed electronic consents are deidentified and stored in a secure database, and all other data is deidentified and kept in a REDCap database stored on University of Sydney servers, accessible only by the study team. Any identifiable information will be stored separately and securely. At completion of the study, all source material will be archived for at least 15 years and then shredded. Deidentified electronic data will be retained at the University of Sydney for at least 15 years and then permanently deleted.

## Discussion

This is a prospective, multicentre RCT evaluating a tailored self-management package in people with fibrotic ILD over 12 weeks with follow-up at 26 and 52 weeks. It aims to assess whether the intervention improves HRQoL, reduces symptoms, increases physical activity and self-efficacy levels, and influences treatment choice in people with ILD. It will also explore if the intervention is cost-effective and implementable.

REBUILD-SM provides evidence-based content via the REBUILD app and self-management modules, leveraging existing technology to maximise reach while minimising costs relative to more conventional face-to-face interventions. Features with proven efficacy in enhancing usage rates have been employed, such as human health coaching to increase motivation and adherence; [37] personalised support from a healthcare professional during the intervention; [38] and automated reminders to encourage reengagement and data completion [39]. 

Recent studies in patients with ILD have demonstrated both feasibility and acceptability of remote monitoring, including oxygen saturation levels and lung function through home spirometry, [40–42] as well as virtual exercise programs [43]. To our knowledge, no studies to date have evaluated a health professional-supported self-management package in this group.

The REBUILD-SM intervention has the potential to improve outcomes for patients with ILD, increase their HRQoL, and instil a greater sense of control through enhanced symptom and side effect management, access to up-to-date educational resources, knowledge of available treatments, and access to clinical trial opportunities. It is hoped that better ability to self-manage will reduce healthcare utilisation and associated costs for patients (particularly those residing rurally or remotely) and the healthcare system.

This study is novel in its approach to clinical trial delivery, as to our knowledge, itis the first ILD-specific app to be evaluated for providing the platform for delivery of a clinical trial intervention. We have chosen to run this trial using the REBUILD app, including obtainment of consent, randomisation, and endpoint collection to reduce the burden of clinical trial participation on patients through what is anticipated to be a streamlined and user-friendly experience. It is hoped that this platform will also reduce the burden of clinical trial coordination on sites, with the potential for this trial to provide a reference for future studies in the field.

A potential limitation of this study is that recruitment is via tertiary ILD centres only, as participants may already have good self-management at baseline due to the provision of education and support by the nurses attached to their centre. The requirement for digital literacy may also bias recruitment towards younger participants with less severe disease, as this has been noted in other mHealth research [44]. To help mitigate this, patients with ILD across the full age and disease severity spectrum will be approached for study participation and advised that technical support will be available throughout the intervention period. Another limitation is that at present, the app and self-management package are only available in English, limiting the generalisability of findings to English speaking populations. If found to be of benefit in the study cohort, planned translation of the digital resources into other languages would enable broader evaluation.

## Conclusion

Many suffering with ILD have debilitating symptoms and associated poor HRQoL. It is hoped that this digital intervention will improve HRQoL for participants through better self-management, increase autonomy, reduce symptoms, and minimise healthcare spending. If successful, the findings have the potential to shape clinical practice and inform future studies in the field by harnessing the broadly accessible, dynamic, and scalable aspects of digital health technology.

## Data Availability

The data generated during this study will be stored in a secure repository and may be made available upon reasonable request.

## Abbreviations

AILDR: Australasian Interstitial Lung Disease Registry
COPD: Chronic obstructive pulmonary disease
ED: Emergency Department
eHEALS: eHealth and Literacy scale
GSE: General Self-Efficacy scale
HADS: Hospital Anxiety and Depression scale
HCCQ: Healthcare Climate questionnaire
HRQoL: Health-related quality of life
ILD: Interstitial lung disease
IPF: Idiopathic pulmonary fibrosis
K-BILD: King’s Brief Interstitial Lung Disease questionnaire
MAUQ: mHealth App Usability questionnaire
MBS: Medicare Benefits Schedule
PBS: Pharmaceutical Benefits Scheme
PM_2.5_: Particulate matter with a diameter ≤ 2.5 micrometres
PROBE: Prospective Randomised Open Blinded Endpoint
QALY: Quality-adjusted life-years
RCT: Randomised controlled trial
